# Selection of the surgical approach for patients with cStage IA lung squamous cell carcinoma: A population-based propensity score matching analysis

**DOI:** 10.3389/fonc.2022.946800

**Published:** 2022-08-23

**Authors:** Shengteng Shao, Guisong Song, Yuanyong Wang, Tengfei Yi, Shuo Li, Fuhui Chen, Yang Li, Xiaotong Liu, Bin Han, Yuhong Liu

**Affiliations:** Department of Thoracic Surgery, The Affiliated Hospital of Qingdao University, Qingdao, China

**Keywords:** cStage IA lung squamous cell carcinoma, survival, segmentectomy, wedge resection, lobectomy, propensity score matching, SEER

## Abstract

**Background:**

This study aimed to conduct a comparative analysis of the survival rates after segmentectomy, wedge resection, or lobectomy in patients with cStage IA lung squamous cell carcinoma (SCC).

**Methods:**

We enrolled 4,316 patients who had cStage IA lung SCC from the Surveillance, Epidemiology, and End Results (SEER) database. The Cox proportional hazards model was conducted to recognize the potential risk factors for overall survival (OS) and lung cancer-specific survival (LCSS). To eliminate potential biases of included patients, the propensity score matching (PSM) method was used. OS and LCSS rates were compared among three groups stratified according to tumor size.

**Results:**

Kaplan–Meier analyses revealed no statistical differences in the rates of OS and LCSS between wedge resection (WR) and segmentectomy (SG) groups for patients who had cStage IA cancers. In patients with tumors ≤ 1 cm, LCSS favored lobectomy (Lob) compared to segmentectomy (SG), but a similar survival rate was obtained for wedge resection (WR) and lobectomy (Lob). For patients with tumors sized 1.1 to 2 cm, lobectomy had improved OS and LCSS rates compared to the segmentectomy or wedge resection groups, with the exception of a similar OS rate for lobectomy and segmentectomy. For tumors sized 2.1 to 3 cm, lobectomy had a higher rate of OS or LCSS than wedge resection or segmentectomy, except that lobectomy conferred a similar LCSS rate compared to segmentectomy. Multivariable analyses showed that patients aged ≥75 and tumor sizes of >2 to ≤3 cm were potential risk factors for OS and LCSS, while lobectomy and first malignant primary indicator were considered protective factors. The Cox proportional analysis also confirmed that male patients aged ≥65 to <75 were independent prognostic factors that are indicative of a worse OS rate.

**Conclusions:**

The tumor size can influence the surgical procedure recommended for individuals with cStage IA lung SCC. For patients with tumors ≤1 cm, lobectomy is the recommended approach, and wedge resection or segmentectomy might be an alternative for those who cannot tolerate lobectomy if adequate surgical margin is achievable and enough nodes are sampled. For tumors >1 to ≤3 cm, lobectomy showed better survival outcomes than sublobar resection. Our findings require further validation by randomized controlled trial (RCT) or other evidence.

## Introduction

Lung cancer is the leading cause of cancer-related mortality worldwide, with non-small cell lung cancer (NSCLC) accounting for over 85% of cases (1). As one of the major pathological types of NSCLC, squamous cell carcinoma (SCC) accounts for about 30% (2). With the increasing use of low-dose helical computed tomography (CT) and high-resolution CT (HRCT) for lung cancer screening and diagnosis, a growing number of patients are diagnosed earlier (3, 4), the majority of whom are non-smokers with small-sized peripheral lung adenocarcinomas (ADCs). Meanwhile, the number of patients with early-stage SCC is also increasing gradually (5).

For early-stage NSCLC, stereotactic ablative radiotherapy (SBRT) does not achieve surgically equivalent oncological outcomes (6) and is recommended for patients who are medically inappropriate for surgery (7). Surgery is still regarded to be the mainstay treatment for patients with early-stage NSCLCs (8, 9). Lobectomy plus lymph node removal has been acknowledged as standard treatment for stage I NSCLC since the randomized controlled trials (RCTs) conducted by the Lung Cancer Study Group in 1995 (10). Additionally, lobectomy not only has lower regional and distant recurrence rates, but also has better survival outcomes compared with wedge resection or segmentectomy in stage I NSCLC (11). However, patients who cannot undergo lobectomy due to old age, poor lung function, or other preoperative comorbidities often need to undergo a limited resection instead (12–14). Currently, wedge resection (WR) and segmentectomy (SG) have become important treatment strategies for patients with stage IA NSCLCs (14, 15). Moreover, segmentectomy is widely used in small-sized NSCLC tumors (16, 17) and has been reported to achieve similar long-term survival benefits as patients that received lobectomy (15, 18).

Several studies have investigated the appropriate surgical procedures for early-stage lung ADC (19) and NSCLC. However, no specific research comparing the survival outcomes of segmentectomy, wedge resection, and lobectomy in patients with cStage IA SCCs is currently available. To that end, we utilized the SEER database to analyze and compare the survival rates of patients with cStage IA SCC receiving different treatment approaches to provide more insight into the optimal surgical strategy for cStage IA SCC based on tumor size.

## Methods

### Patient population

The patients in this research were extracted from the Surveillance, Epidemiology, and End Results (SEER) database, a population-based cancer database that provides information on cancer incidence in 18 registries of the United States and covers about 30% of the population. We identified all individuals with cStage IA (T1N0M0) lung SCC (SEER codes 8052, 8070–8075, 8083, 8084, and 8123) who were verified by pathology and had undergone wedge resection, segmentectomy, or lobectomy (SEER codes were 21, 22, and 30 to 33, respectively) from January 2010 to December 2015. Patients were not eligible if they had received chemotherapy or radiation prior to, during, or after the surgical treatment or if the baseline characteristics were unknown.

In this retrospective study, the information of demography (age, gender, marital status, and race), characteristics of the tumor (primary site, laterality, differentiation, and size), treatment (surgical procedure, chemotherapy, and radiotherapy), the cause of death, and first malignant primary indicator were collected from the SEER database. Based on the surgical approach received, patients were separated into three groups: wedge resection (WR), segmentectomy (SG), and lobectomy (Lob).

### Outcomes

We defined overall survival (OS) and lung cancer-specific survival (LCSS) using the codes provided by the SEER database. The OS rate was the primary endpoint in our research. This was calculated from the surgery date to the date of the patient’s death from any cause or last follow-up. The secondary endpoint was the LCSS rate, which was calculated from the surgery date to the date of death due to lung cancer. The last follow-up date was 31 December 2018 (time range from 1 to 107 months).

### Statistical analysis

The categorical variables were compared using Pearson’s *χ^2^* test for baseline characteristics. Cox proportional hazards regressions model were performed to identify the potential and independent risk factors affecting the rates of OS and LCSS for cStage IA SCC patients. We divided the eligible patients into three groups according to the surgical approach, namely, lobectomy (Lob), segmentectomy (SG), and wedge resection (WR). According to different outcome events (patient death or loss to follow-up and patient death due to lung cancer) of patients, we performed univariate and multivariate Cox regression analysis to screen out the independent risk factors influencing the OS and LCSS. Significant variables in multivariate analysis were independent risk factors affecting the prognosis of patients. The variables affecting the OS of patients were age, gender, tumor size, surgical method, and first malignant primary indicator, and the variables affecting LCSS of patients were age, tumor size, surgical method, and first malignant primary indicator. Combining the differences in patients’ baseline characteristics and clinical practice, we defined age, gender, the laterality of the tumor, the lobe of the tumor, and first malignant primary indicator as variables used in propensity score matching (PSM).

PSM methods were applied to minimize the potential biases in the basic features between the cases and controls. The patients were separated into three strata according to tumor size (≤1.0 cm, 1.1 to 2.0 cm, and 2.1 to 3.0 cm), and in each stratum, three groups of patients who underwent different surgeries were separately matched in a ratio of 1:1. For example, for individuals with tumors smaller than 1 cm and the outcome event of death or loss to follow-up, 1:1 PSM was performed for segmentectomy versus wedge resection, segmentectomy versus lobectomy, and wedge resection versus lobectomy group, respectively.

The Kaplan–Meier method was used to analyze and compare the rates of OS and LCSS among patients with cStage IA lung SCC of 1 cm or smaller, 1.1 to 2.0 cm, and 2.1 to 3.0 cm receiving segmentectomy, wedge resection, or lobectomy in both the entire cohort and the cohort after PSM.

For statistical results, IBM SPSS 26.0 (SPSS, Inc, Chicago, IL) was used for all analyses, and GraphPad Prism 9.0 (GraphPad Software, San Diego, CA) was used to draw the survival curve. The reported significance levels were two-sided, and statistical significance was defined as the value of *p* ≤ 0.05.

## Results

### Patient characteristics

A total of 4,316 eligible patients with cStage IA lung SCC (≤3 cm) were identified, namely, 254 (5.9%) who received segmentectomy, 1,085 (25.1%) who underwent wedge resection, and 2,977 (69.0%) who had a lobectomy. The median follow-up time was 50.5 months for the entire cohort, 47 months for segmentectomy, 45 months for wedge resection, and 53 months for lobectomy. A total of 2,052 patients died [141 (6.9%) from segmentectomy, 619 (30.1%) from the wedge resection, and 1,292 (63.0%) from the lobectomy groups] and 927 patients suffered from lung cancer-specific deaths [65 (7.0%) from the segmentectomy, 300 (32.4%) from the wedge resection, and 562 (60.6%) from the lobectomy groups].

Our research revealed that sublobar resection (wedge resection or segmentectomy) was operated in patients who were more elderly or had a smaller tumor size, especially if the tumor is ≤2.0 cm. When a patient had only one primary malignant neoplasm, lobectomy or segmentectomy was more likely to be performed. **Table 1** demonstrates the baseline characteristics of the primary cohort.

**Table 1:** Baseline characteristics of patients with stage IA squamous cell lung cancer.

Variables	Segmentectomy (*N* = 254)	Wedge resection (*N* = 1,085)	Lobectomy (*N* = 2,977)	*p*-value
Marital status				0.114
Married	122 (48.0%)	582 (53.6%)	1,641 (55.1%)	
Unmarried	113 (44.5%)	453 (41.8%)	1,193 (40.1%)	
Unknown	19 (7.5%)	50 (4.6%)	143 (4.8%)	
Age (years)				<0.001
<55	2 (0.8%)	23 (2.1%)	119 (4.0%)	
≥55, <65	36 (14.2%)	166 (15.3%)	614 (20.6%)	
≥65, <75	116 (45.7%)	481 (44.3%)	1,385 (46.5%)	
≥75	100 (39.4%)	415 (38.2%)	859 (28.9%)	
Sex				<0.001
Male	107 (42.1%)	578 (53.3%)	1,642 (55.2%)	
Female	147 (57.9%)	507 (46.7%)	1,335 (44.8%)	
Race				0.074
White	219 (86.2%)	972 (89.6%)	2,648 (8.9%)	
Black	30 (11.8%)	74 (6.8%)	233 (7.8%)	
Others	5 (2.0%)	39 (3.6%)	96 (3.2%)	
Primary site				0.016
Upper lobe	148 (58.3%)	694 (64.0%)	1,082 (60.5%)	
Middle lobe	5 (2.0%)	38 (3.5%)	145 (4.9%)	
Lower lobe	101 (39.8%)	353 (32.5%)	1,030 (34.6%)	
Laterality				0.005
Left	134 (52.8%)	488 (45.0%)	1,267 (42.6%)	
Right	120 (47.2%)	597 (55.0%)	1,710 (57.4%)	
Differentiation				0.191
Well	8 (3.1%)	37 (3.4%)	110 (3.7%)	
Moderately	153 (60.2%)	604 (55.7%)	1,614 (54.2%)	
Poorly	93 (36.6%)	436 (40.2%)	1,244 (41.8%)	
No	0 (0%)	8 (0.7%)	9 (0.3%)	
Tumor size (mm)				<0.001
≤10	36 (14.2%)	216 (19.9%)	245 (8.2%)	
>10, ≤20	139 (54.7%)	615 (56.7%)	1,429 (48.0%)	
>20, ≤30	79 (31.1%)	254 (23.4%)	1,303 (43.8%)	
T1 verified by pathology				0.481
Yes	249 (98.0%)	1,073 (98.9%)	2,932 (98.5%)	
No	5 (2.0%)	12 (1.1%)	45 (1.5%)	
N0 verified by pathology				<0.001
Yes	186 (73.2%)	544 (50.1%)	2,797 (94.0%)	
No	68 (26.8%)	541 (49.9%)	180 (6.0%)	
Cause of dead				0.093
Lung cancer	65 (25.6%)	300 (27.6%)	562 (18.9%)	
Chronic pulmonary disease	24 (9.4%)	69 (6.4%)	141 (4.7%)	
Diseases of heart	16 (6.3%)	70 (6.5%)	173 (5.8%)	
Others	36 (14.2%)	180 (16.6%)	416 (14.0%)	
First malignant primary indicator				<0.001
Yes	153 (60.2%)	581 (53.5%)	2,052 (68.9%)	
No	101 (39.8%)	504 (46.5%)	925 (31.1%)	

### Tumors ≤ 1.0 cm

**Figure 1:**
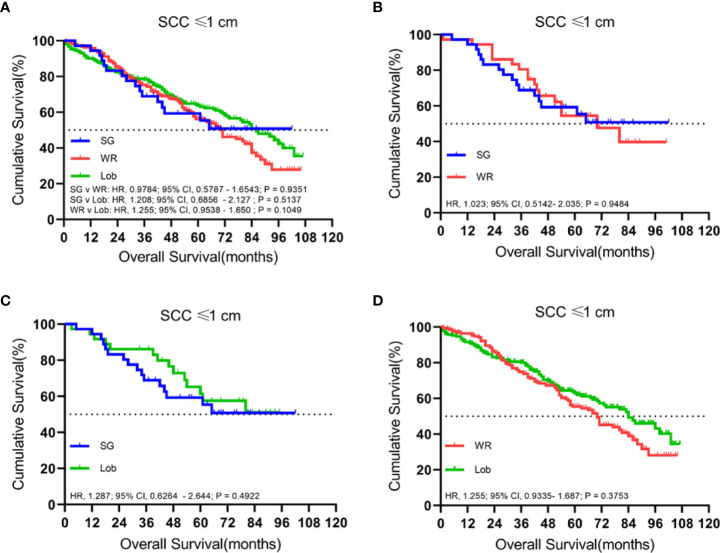
Kaplan–Meier survival curves of overall survival for tumors of ≤1.0 cm in the primary cohort **(A)** and the propensity score-matched cohort: **(B)** SG versus WR (*n* = 36 pairs), **(C)** SG versus Lob (*n* = 36 pairs), and **(D)** WR versus Lob (*n* = 193 pairs).

**Figure 2:**
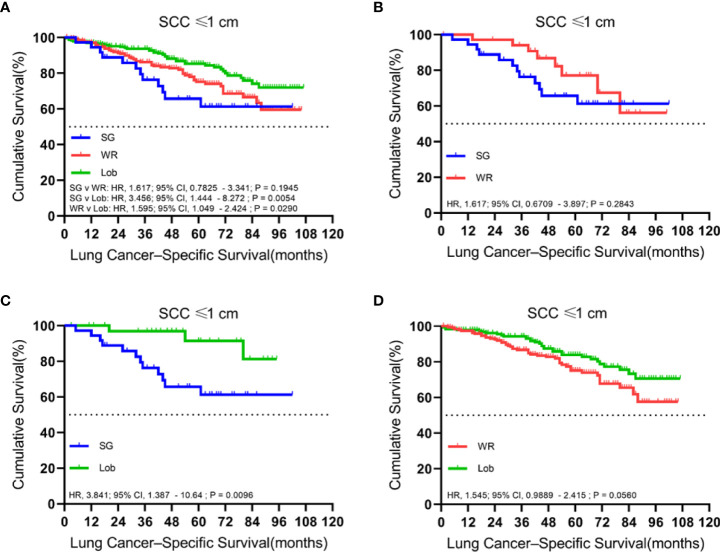
Kaplan–Meier survival curves of lung cancer-specific survival for tumors of ≤1.0 cm in the primary cohort **(A)** and the propensity score-matched cohort: **(B)** SG versus WR (*n* = 36 pairs), **(C)** SG versus Lob (*n* = 36 pairs), and **(D)** WR versus Lob (*n* = 193 pairs).

We identified 497 patients with cStage IA lung SCC with a tumor size of 1.0 cm or smaller. Thirty-six (7.2%) of them underwent segmentectomy, 216 (43.5%) received wedge resection, and 245 (49.3%) had their lobe removed. The median follow-up time was 52 months. OS analysis revealed that the unmatched and propensity-matched results showed no statistical differences in the OS rates among the three treatment groups (**Figure 1**). On the other hand, LCSS analysis revealed that lobectomy achieved a better LCSS than segmentectomy or wedge resection in the unmatched cohort. Interestingly, lobectomy still showed a better LCSS rate when compared with segmentectomy in the matched cohort. However, there was no statistical difference between lobectomy and wedge resection (**Figure 2**).

### Tumors sized 1.1 to 2.0 cm

There were 2,183 patients with cStage IA lung SCC with tumor sizes ranging from 1.1 to 2.0 cm. A total of 1,429 (65.5%) patients received lobectomy, 615 (28.2%) underwent wedge resection, and 139 (6.4%) had a segmentectomy. The median follow-up time was 52 months.

**Figure 3:**
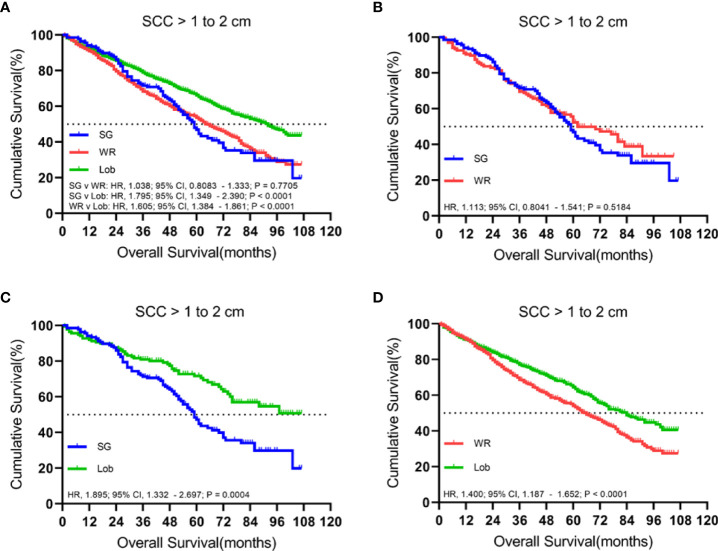
Kaplan–Meier survival curves of overall survival for tumors from 1.1 to 2.0 cm in the primary cohort **(A)** and propensity score-matched cohort: **(B)** SG versus WR (*n* = 139 pairs), **(C)** SG versus Lob (*n* = 138 pairs), and **(D)** WR versus Lob (*n* = 598 pairs).

**Figure 4:**
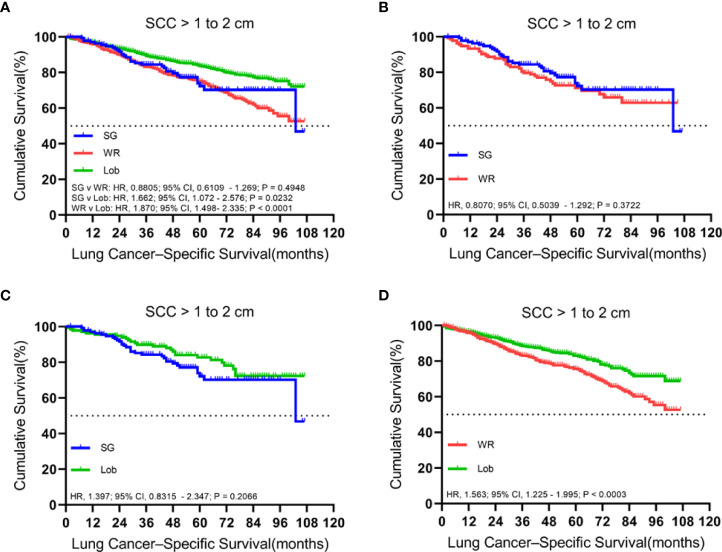
Kaplan–Meier survival curves of lung cancer-specific survival for tumors from 1.1 to 2.0 cm in the primary cohort **(A)** and the propensity score-matched cohort: **(B)** SG versus WR (*n* = 139 pairs), **(C)** SG versus Lob (*n* = 138 pairs), and **(D)** WR versus Lob (*n* = 598 pairs).

In both unmatched and matched cohorts, lobectomy was found to be superior to segmentectomy or wedge resection in terms of OS (**Figure 3**). Meanwhile, LCSS analysis showed that lobectomy was better than segmentectomy or wedge resection in the unmatched cohort. In contrast, lobectomy and segmentectomy displayed no significant difference in the matched cohort (**Figure 4**).

### Tumors sized 2.1 to 3.0 cm

**Figure 5:**
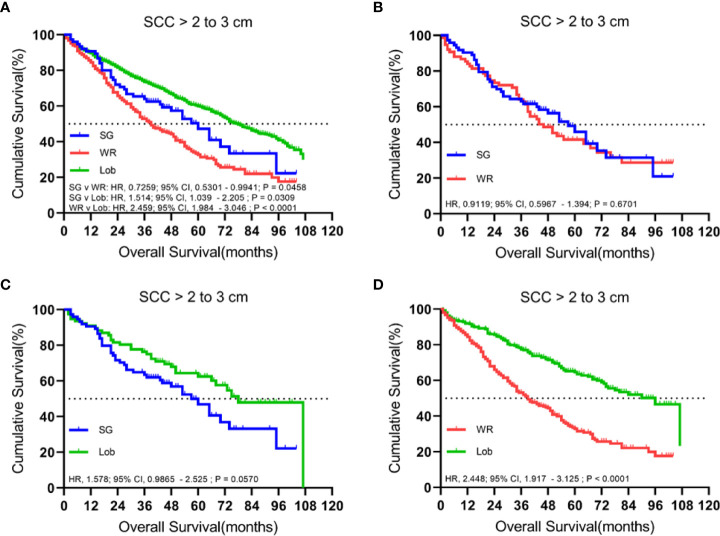
Kaplan–Meier survival curves of overall survival for tumors from 2.1 to 3.0 cm in the primary cohort **(A)** and the propensity score-matched cohort: **(B)** SG versus WR (*n* = 77 pairs), **(C)** SG versus Lob (*n* = 78 pairs), and **(D)** WR versus Lob (*n* = 253 pairs).

**Figure 6:**
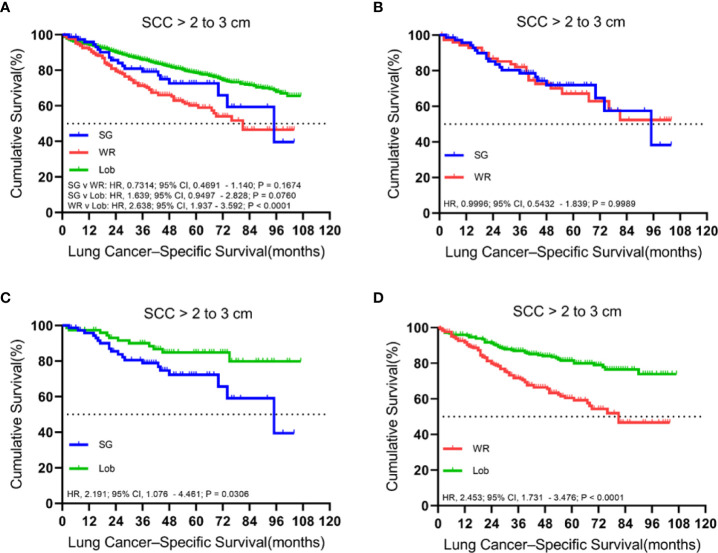
Kaplan–Meier survival curves of lung cancer-specific survival for tumors from 2.1 to 3.0 cm in the primary cohort **(A)** and the propensity score-matched cohort: **(B)** SG versus WR (*n* = 77 pairs), **(C)** SG versus Lob (*n* = 78 pairs), and **(D)** WR versus Lob (*n* = 253 pairs).

A total of 1,636 patients were identified with cStage IA lung SCC with a tumor size of 2.1 to 3.0 cm who underwent segmentectomy (79; 4.8%), wedge resection (254; 15.5%), or lobectomy (1,303; 79.6%). The median follow-up time was 48.5 months. OS analysis revealed that segmentectomy was associated with a superior OS compared to wedge resection but had an inferior OS rate in comparison to lobectomy in the unmatched cohort. Similarly, a better OS rate was observed for those patients who had undergone a lobectomy rather than a wedge resection in the matched cohorts (**Figure 5**). Importantly, lobectomy had a better LCSS rate than wedge resection in both unmatched and matched cohorts. In addition, lobectomy achieved a better LCSS than segmentectomy in the matched cohorts (**Figure 6**).

### Cox regression analysis

We used the Cox proportional hazards regressions model to identify the potential risk factors correlating with OS and LCSS in cStage IA SCC patients (**Table 2**). Univariate Cox regression analysis showed that age, surgical procedure, tumor size, and first malignant primary indicator were significantly correlated with OS and LCSS. Moreover, OS was also found to be associated with gender.

**Table 2:** Cox proportional hazards regression model for overall survival and lung cancer-specific survival in patients with stage IA squamous cell lung cancer.

Variables	OS				LCSS		
	Univariable analysis	Multivariable analysis			Univariable analysis	Multivariable analysis		
	*p*	HR	95% CI	*p*	*p*	HR	95% CI	*p*
Marital status	0.171				0.245			
Married								
Unmarried								
Unknown								
Age (years)	<0.001				<0.001			
<55		1	(Reference)			1	(Reference)	
≥55, <65		1.258	0.923–1.716	0.146		0.973	0.644–1.469	0.896
≥65, <75		1.546	1.149–2.080	0.004		1.195	0.810–1.765	0.369
≥75		2.146	1.593–2.891	<0.001		1.508	1.018–2.234	0.041
Sex	<0.001				0.052			
Male		1	(Reference)					
Female		0.773	0.707–0.844	<0.001				
Race	0.823				0.630			
White								
Black								
Others								
Primary site	0.242				0.103			
Upper lobe								
Middle lobe								
Lower lobe								
Laterality	0.385				0.927			
Left								
Right								
Differentiation	0.505				0.233			
Well								
Moderately								
Poorly								
No								
Surgical approach	<0.001				<0.001			
Segmentectomy		1	(Reference)			1	(Reference)	
Wedge resection		1.011	0.841–1.214	0.909		1.098	0.839–1.437	0.496
Lobectomy		0.674	0.565–0.803	<0.001		0.651	0.503–0.844	0.001
Tumor size (mm)	0.001				0.002			
≤10		1	(Reference)			1	(Reference)	
>10, ≤20		1.035	0.896–1.195	0.642		1.064	0.857–1.320	0.577
>20, ≤30		1.344	1.148–1.550	<0.001		1.509	1.206–1.888	<0.001
First malignant primary indicator	<0.001				<0.001			
Yes		1	(Reference)			1	(Reference)	
No		1.263	1.154–1.381	<0.001		1.312	1.149–1.499	<0.001

Furthermore, all variables with a *p*-value less than 0.05 were included in the multivariate analysis. We found that patients aged ≥ 75 with a tumor size of >2.0 to ≤3.0 cm were negatively correlated to OS and LCSS, while the lobectomy and first malignant primary indicator were considered to be protective factors. Additionally, we found that the male gender and those aged ≥65 to <75 were independent factors for poor OS while there was no statistical significance for cancer-specific survival.

## Discussion

The Lung Cancer Study Group reported that lobectomy could achieve a better OS and lower local recurrence rate than sublobar resection in the treatment of stage I NSCLC after a randomized prospective multi-institutional controlled trial in 1995 (10). Since then, lobectomy with lymph node dissection has been implemented as the standard for resectable early-stage NSCLC (8, 20). However, with the recent improvements in screening methods and surgical techniques, there is a growing body of evidence showing that sublobar resection is comparable to lobectomy in terms of surgical outcomes.

Dai et al. and Cao et al. previously corroborated on the use of the recommended surgical option for early-stage NSCLC based on tumor size (15, 18). However, they found that the SCC subtype showed significant differences in terms of clinicopathological and genetic features compared to the ADC subtype, showing a worse clinical outcome for early-stage lung cancer patients (21). Moreover, Li et al. found that segmentectomy was superior to wedge resection in patients with stage IA SCC, but the prognosis of wedge resection and segmentectomy were roughly equivalent in stage IA ADC patients (22). Several studies have been carried out to determine the appropriate surgical approach for small-sized SCC. For instance, Chen et al. compared the survival rate after patients with stage I lung SCC with a tumor size ≤ 3 cm received sublobar resection or lobectomy (23). Herein, we attempted to investigate the effectiveness of three surgical approaches (segmentectomy, wedge resection, and lobectomy) in cStage IA SCC. Importantly, we included a larger number of patients compared to Chen et al.’s study.

Previous studies have shown that patients with NSCLC benefit more from segmentectomy than wedge resection. For instance, Dai et al. concluded that segmentectomy is supposed to be suggested for NSCLC patients who are not candidates for lobectomy (15). Hou et al. found that segmentectomy achieved a better survival rate than wedge resection in stage I NSCLC (24). Additionally, Reveliotis et al. identified that segmentectomy is better than wedge resection on the aspects of the rates of regional recurrence and cancer-related mortality (25). However, there are also conflicting reports. Several retrospective studies reported that wedge resection might be ontologically equivalent to segmentectomy in patients with tumors ≤ 1 cm (18, 26). Moreover, a meta-analysis has identified that WR and SG might be comparable in select patients with early-stage lung cancer, especially for tumors sized 2 cm or smaller (27). In addition, a prospective randomized trial (ACOSOG Z4032) by Sybron Harrison et al. supported the view that wedge resection is comparable to segmentectomy (28). Our study analyzed the prognosis of segmentectomy versus wedge resection in cStage IA SCC patients on the basis of the eighth TNM classification. We found that the survival difference was not significant between segmentectomy and wedge resection for tumors of T1a (≤1 cm), T1b (>1 to 2 cm), and T1c (>2 to 3 cm) (29). In contrast, Li et al. discovered that segmentectomy outperformed wedge resection in terms of survival for patients with stage IA SCC (22), but their study sample was considerably smaller than ours, and no subgroup analysis of SCC stratified by tumor size was performed.

Lobectomy is commonly accepted as being better than wedge resection for patients with stage I NSCLC (30). In clinical practice, wedge resection is usually performed in patients with poor lung function or those with other comorbidities that might not be suitable for lobectomy (14). A study using the SEER database also reported that Lob showed better survival rates than WR for NSCLC of ≤ 2 cm (15). However, several studies suggested that no significant difference was found in survival outcome among patients with stage IA NSCLC sized ≤ 1 cm who underwent lobectomy and wedge resection (31, 32). Our study obtained the same result for cStage IA SCC ≤ 1 cm and identified that lobectomy was superior to wedge resection for cStage IA SCC > 1 to 3 cm.

As for segmentectomy versus lobectomy in cStage IA SCC ≤ 1 cm, patients showed similar OS, but lobectomy was superior to segmentectomy in terms of LCSS rate. This may be attributed to the intraoperative assessment of lymph node metastasis and adequate surgical margin (33). SG has adequate surgical margin to achieve a successful resection of peripheral small-sized SCC. However, for some nodules, standard segmentectomy cannot achieve a safe margin distance, which could result in a worse clinical outcome (34). Therefore, we recommend lobectomy as the surgical procedure for patients with cStage IA SCC ≤ 1 cm. For those who cannot tolerate lobectomy (advanced age, poor lung function, previous lung surgery, or other serious comorbidities), WR or SG might be the proper treatment under the premise of sufficient surgical margin and lymph node sampling.

The appropriate surgical procedures for stage IA NSCLC have been discussed in several papers. They discovered that for tumors measuring ≤ 1.0 cm and between 1.1 and 2.0 cm, lobectomy and segmentectomy have identical survival outcomes (18, 27). A meta-analysis has also suggested that segmentectomy was potentially feasible for NSCLC ≤ 2 cm (35). Results from a recently released Phase 3 clinical trial (JCOG0802/WJOG4607L) confirm the above conclusions (36). However, most of the patients included in JCOG0802 were peripheral ADCs. Whether this conclusion is suitable for small lung SCC remains to be investigated. Also, lobectomy is considered superior to segmentectomy for tumors sized between 2.1 and 3.0 cm (15, 18, 27). In our study, we found that lobectomy showed a better OS rate than segmentectomy for cStage IA SCC >1 to ≤2 cm, but not for those >2 to ≤3 cm. As for LCSS rate, there was no statistical difference for tumors >1 to ≤2 cm between lobectomy and segmentectomy. Patients with cStage IA SCC >2 to 3 cm may benefit from lobectomy with a lower risk of cancer-related death. In addition, lobectomy was superior to WR for cStage IA SCC >1 to 2 cm and >2 to 3 cm. Therefore, we conclude that for patients with cStage IA SCC sized >1 to 2 cm or >2 to ≤3 cm, the conventional surgical approach may still be lobectomy, while segmentectomy could be an alternative approach for those not suitable for lobectomy.

In the Cox proportional analysis, apart from surgical procedures, we also verified other independent prognostic factors in node-negative SCC. Our retrospective study showed that patients aged ≥65 and ≥75 are at higher risk for worse OS and LCSS, respectively. The male gender was revealed to be a risk factor correlated to the OS rate, while the LCSS rate was not significantly influenced by gender. Several studies also confirmed that age and gender were validated factors for predicting personal survival rate (37). Tumor size may correspond with the appropriate surgical procedure on those patients with early-stage SCC (18, 23). The results of our study revealed that a tumor size of >2 to ≤3 cm may pose a risk for OS and LCSS in comparison to those smaller than 2 cm, and that a lobectomy procedure was considered to be a protective factor for patients with cStage IA SCC sized >2 to ≤3 cm. In addition, one stage IA SCC individual with two or more histologically distinct malignancies had a worse OS and LCSS, while the first malignant primary indicator was found to be an independent factor synonymous with a good survival outcome.

Nevertheless, there are several limitations to our study. Firstly, this is a retrospective study, and all the data were collected from the SEER database. Although we attempted to balance the baseline characteristics of the patients using the propensity score-matched method, there are some inevitable inherent biases. Secondly, in some cases, where a total lobectomy is not feasible, wedge resection or segmentectomy may be an effective treatment, especially for elderly patients, those with severe impairments in lung function, or others (12–14). However, the comorbidities and pulmonary function data were not included in the SEER database. Thirdly, the SEER database did not provide tumor location data (central or peripheral). Sung Ye et al. previously uncovered that peripheral SCC has different clinicopathological and genetic features compared to the central type (38), showing a significantly better disease-free survival (DFS) and OS (39). Lastly, the SEER database did not provide other important information, such as detailed surgical records (open or minimally invasive, intentional segmentectomy or not, lymph node sampling or mediastinal lymph node dissection) and imaging appearance of tumor (the imaging size of the tumor, solid component proportion), to name a few.

## Conclusion

In conclusion, for patients with cStage IA SCC sized ≤ 1 cm, lobectomy is more advantageous in improving their cancer-specific survival and may be the standard procedure. WR and SG are found to be comparable in terms of OS and recommended for those who cannot tolerate lobectomy. For tumors >1 to ≤2 cm or >2 to ≤3 cm, our study revealed that lobectomy showed better survival outcomes compared to sublobar resection. Therefore, lobectomy is supposed to be performed for those patients, while segmentectomy may be an adequate alternative. The conclusions in this article still need more evidence to be further confirmed. For example, RCTs on cStage IA SCC, or PSM with more treatment details may lead to more convincing conclusions.

## Data availability statement

The data from the SEER database is free and publicly available. Further inquiries can be directed to the corresponding author.

## Ethics statement

Ethical review and approval was not required for the study on human participants in accordance with the local legislation and institutional requirements. Written informed consent for participation was not required for this study in accordance with the national legislation and the institutional requirements.

## Author contributions

YHL and SS conceived and designed the study. YL, XL, and BH collected the data. SS and GS analyzed the data. SS, YW, TY, SL, and FC wrote this manuscript. All authors contributed to the article and approved the submitted version.

## Acknowledgments

The authors are grateful for the invaluable support and useful discussions with other members of the Department of Thoracic Surgery, and thank Home for Researchers (www.home-for-researchers.com) for language enhancement of this paper.

## Conflict of interest

The authors declare that the research was conducted in the absence of any commercial or financial relationships that could be construed as a potential conflict of interest.

## Publisher’s note

All claims expressed in this article are solely those of the authors and do not necessarily represent those of their affiliated organizations, or those of the publisher, the editors and the reviewers. Any product that may be evaluated in this article, or claim that may be made by its manufacturer, is not guaranteed or endorsed by the publisher.
